# A nanoparticle-based approach to improve the outcome of cancer active immunotherapy with lipopolysaccharides

**DOI:** 10.1080/10717544.2018.1469684

**Published:** 2018-06-14

**Authors:** Maryam A. Shetab Boushehri, Mona M. A. Abdel-Mottaleb, Arnaud Béduneau, Yann Pellequer, Alf Lamprecht

**Affiliations:** a Department of Pharmaceutics, University of Bonn, Bonn, Germany;; b Laboratory of Pharmaceutical Engineering (EA4267), University of Franche-Comté, Besançon, France;; c Department of Pharmaceutics and Industrial Pharmacy, Faculty of Pharmacy, Ain Shams University, Cairo, Egypt

**Keywords:** Lipopolysaccharide (LPS), nanoparticles, pathogen-mimicking properties, localized necrosis, cancer immunotherapy

## Abstract

This study sought to develop a simple nanoparticle-based approach to enhance the efficiency and tolerability of lipopolysaccharide (LPS), a potent ligand of Toll-like Receptor 4 (TLR4), for immunotherapy in cancer. Despite holding promise within this context, the strong pro-inflammatory properties of LPS also account for its low tolerability given localized and systemic side effects, which restrict the administrable dosage. Herein, we investigated the effect of LPS decoration as a surface-active molecule on a polymeric matrix upon its efficiency and tolerability. The LPS-decorated nanoparticles (LPS-NP) were about 150 nm in size, with slightly negative zeta potential (about −15 mV) and acceptable LPS incorporation (about 70%). *In vitro*, the particles accounted for a higher induction of apoptosis in tumor cells cultured with murine splenocytes compared to LPS solution. When used for the treatment of a murine syngeneic colorectal tumor model, higher intratumoral deposition of the particle-bound LPS was observed. Furthermore, unlike LPS solution, which accounted for localized necrosis at high concentrations, treatment of tumor-bearing animals with equivalent doses of LPS-NP was well tolerated. We propose that the observed localized necrosis can be Shwartzman phenomenon, which, due to modulated 24-h post-injection systemic TNF-α and LPS concentrations, have been avoided in case of LPS-NP. This has in turn enhanced the therapeutic efficiency and enabled complete tumor regression at concentrations at which LPS solution was intolerable. The findings indicate that nanoparticles can serve as beyond carriers for the delivery of superficially decorated LPS molecules, but impact their overall efficiency and tolerability in cancer therapy.

## Introduction

Toll-like Receptor (TLR) agonists have been long since proven promising for reversing the tumor-induced ‘immune-privileged’ attenuation of the body’s immune response (Mellman et al., 2011; Beatty & Gladney, 2015). One of the earliest candidates in this category has been the potent ligand of TLR4, bacterial lipopolysaccharide (LPS), the use of which dates back to the 1940s, when it was first identified as the immunotherapeutic component of Coley’s toxin (Wiemann & Starnes, 1994). Immunotherapy with LPS accounts for the induction of a plethora of pro-inflammatory cytokines and the upregulation of co-stimulatory molecules in antigen presenting cells (APCs). The overall outcome of this turn of events in the presence of the tumor antigens is primarily the generation of a Th1 immune response and the breakage of the tumor-induced immune tolerance (McAleer & Vella, 2008; Awasthi, 2014). Nevertheless, despite having undergone two phases of clinical trials, the anticancer efficiency of intravenously injected LPS has been limited due to significant side effects restricting the administrable dosage (Engelhardt et al., 1991; Otto et al., 1996). Thus, approaches to decrease the LPS-related side effects while maintaining, or even improving, the compound’s pro-inflammatory properties have been long since sought after.

Different strategies have been proposed to fulfill the above-mentioned goal. These include using alternative routes for LPS administration (e.g. intratumoral or intradermal injection) (Goto et al., 1996; Chicoine et al., 2001), synthesis of less toxic LPS derivatives/analogs (Madonna et al., 1986; Sato et al., 1995; de Bono et al., 2000; Roy et al., 2010; Isambert et al., 2013; Matzner et al., 2016), and combination therapy with synergistic immunotherapeutic compounds (Stier et al., 2013; Ando et al., 2015). These approaches, though offering certain advantages, are each associated with shortcomings of its own. For instance, intradermal LPS administration enables a sustained LPS release in the bloodstream, alleviating thereby the associated systemic side effects (Goto et al., 1996). However, it fails to control the localized adverse effects (e.g. localized necrotic reactions) and is thus inappropriate for the administration of high doses. The intratumoral injection allows for the activation of the intratumoral suppressed immune cells (Chicoine et al., 2001), though the dense cellular package and the presence of extracellular matrix restrict the penetration of the molecules from the injection site throughout the tumor tissue (Nichols & Bae, 2012). Development of less toxic LPS derivatives/analogs has been perhaps the most successful strategy, having fruited as the FDA approved monophosphoryl-lipid A (MPLA) as vaccine adjuvant. Nevertheless, maintaining the immunological properties of the developed compounds while reducing the side effects still poses a challenge (Vacchelli et al., 2012). For instance, even though monotherapy with MPLA has been significantly better tolerated when compared to LPS, the results of the clinical trials have shown no direct objective antitumor activity in case of the former (Vosika et al., 1984). Finally, although co-administration of LPS with synergistic immunostimulators is beneficial, few of such agents have been hitherto identified (Barratt et al., 1991; Held et al., 1999).

Within this study, we sought to develop a simple nanotechnology-based approach to address this relatively old problem, i.e. to improve the outcome of LPS-mediated active immunotherapeutic eradication of solid tumors. Conventionally, nanoparticles have ample to offer when used as drug carriers. These include resolving solubility issues, increasing intracorporeal stability, possibility of cellular and intracellular targeting, and controlling the release or cellular uptake of the incorporated cargo, along with the reduction of the associated side effects by hindering the payload from going astray (Merisko-Liversidge & Liversidge, 2008; Paillard et al., 2010).

Due to their amphiphilic nature (Aurell & Wistrom, 1998), LPS molecules possess surface-active properties exploitable for interface stabilization and hence nanoparticle formulation. When used within this context, the LPS molecules will orient themselves upon the interface of the nanoparticles and the surrounding aqueous medium, and enable the formation of LPS-decorated nanoparticles with pathogen mimicking properties (Heinz et al., 2017). Furthermore, nanoparticle surface has been shown to retain amphiphilic drugs, which can minimize their burst release particularly under non-sink conditions, i.e. when injected subcutaneously or intramuscularly (Lamprecht et al., 2002). This could potentially alleviate the side effects associated with the abrupt exposure of the body to high concentrations of potent immunostimulators such as LPS.

With such possibilities in mind, the structural surfactant properties of the LPS molecule along with its TLR4 agonistic characteristics was exploited to develop pathogen-mimicking LPS-decorated poly(lactic-co-glycolic acid) (PLGA)-based nanoparticles (LPS-NP). The particles were then compared to free LPS solution of equivalent dosage in terms of the efficiency and the severity of adverse effects both in cell culture and in animal models.

## Materials and methods

### Materials

LPS from *Salmonella enterica abortus equi* was purchased from Sigma-Aldrich (Stammheim, Germany). PLGA (Rosemere RG 502 H) was obtained from Evonik Röhm GmbH (Darmstadt, Germany). Thiazolyl blue tetrazolium boromide (MTT) and Nile Red were supplied by Sigma-Aldrich. Ethyl acetate and polyethylene glycol 400 were obtained from Fischer Scientific and Caesar & Loretz GmbH (Hilden, Germany), respectively. All other chemicals were of analytical grade.

### Cell lines

Murine colon adenocarcinoma C26 and glioma GL261 cell lines were obtained from National Cancer Institute (Frederick, MD). Murine macrophage RAW264.7 (ATCC^®^ TIB-71^™^) and JAWS II (ATCC^®^ CRL-11904^™^) dendritic cell lines were purchased from American Type Culture Collection (ATCC, Middlesex, United Kingdom). RAW264.7 and C26 cells were grown in RPMI-1640 medium supplemented with 10% FBS, 50 µg/mL streptomycin, 50 U/mL penicillin G, and 2 mM L-glutamine. A medium with similar composition but containing 4 mM L-glutamine was used for the growth of GL261 cells. JAWS II cells were kept in alpha minimum essential medium (α-MEM) with ribonucleosides and deoxyribonucleosides supplemented with 20% FBS, 50 µg/mL streptomycin, 50 U/mL penicillin G, 4 mM L-glutamine, 1 mM sodium pyruvate, and 2.5 µg/mL granulocyte-macrophage colony stimulating factor (GM-CSF). All the cell lines were cultivated in a 37 °C incubator with 5% CO_2_ and 95% humidified air.

Splenocytes were isolated from 6-week-old male BALB/c mice and kept in α-MEM with ribonucleosides and deoxyribonucleosides supplemented with 50 µg/mL streptomycin, 50 U/mL penicillin G, 4 mM L-glutamine, 1 mM sodium pyruvate, and 2.5 µg/mL GM-CSF during experimentation.

### Animals

6-week-old male BALB/c mice were obtained from Janvier Labs (Roubaix, France). The animals were kept at room temperature (25 ± 2 °C) and relative humidity (40–60%) under a 12 h light/dark cycle. Food and water were provided *ad libitum*. All studies were approved by the Institutional Animal Care and Use Committee of the University of Franche-Comté and were carried out in accordance with the recommendations in the Guide for the Care and Use of Laboratory Animals in France.

### Particle preparation and characterization

LPS-NPs were prepared through a simple oil in water emulsification/solvent evaporation technique. Briefly, 10 mg PLGA dissolved in 1 mL ethyl acetate was poured into 2 mL of the aqueous phase, containing 1 mg/mL of LPS as surfactant. The obtained coarse emulsion was then subjected to high sheer using ultrasonic cell disruptor (Bandelin Sonopuls, Berlin, Germany) with 50% power for 1 min, followed by the removal of ethyl acetate under reduced pressure. Labeled nanoparticles for CLSM studies were prepared with Nile Red (25 µg/mL) and FITC-conjugated LPS (50 µg/mL, Sigma-Aldrich). LPS-free PLGA nanoparticles were prepared through a modified solvent displacement method (Ali & Lamprecht, 2013). Briefly, 150 mg PLGA dissolved in 3 mL polyethylene glycol 400 was added dropwise to 30 mL of deionized water at 37 °C and under constant stirring at 400 rpm. The particles were subsequently washed to dispose of excess polyethylene glycol. Following preparation, both blank and LPS-NP were characterized in terms of particle size and zeta potential, using photon correlation spectroscopy and electrophoretic laser Doppler anemometry, respectively. Particle size was measured in terms of effective diameter and PDI using particle size/zeta analyzer (Brookhaven Instruments, Holtsville, NY) at a fixed angle of 90° at 25 °C. For the measurement of zeta potential, nanoparticle suspension was diluted with 10^5^ M sodium chloride solution to adjust the conductivity at 50 μS/cm. Zeta potential was measured at 25 °C, and the error was calculated as the standard deviation (SD) of three independent measurements. The shape and morphological characteristics of the prepared nanoparticles was studied using SEM (Hitachi S-2460N, Hitachi Ltd. Corporation, Tokyo, Japan).

LPS incorporation within the particle structure was indirectly ascertained through the quantification of the free LPS within the supernatant following the centrifugal isolation of the nanoparticles from the suspension (15,000 *g* at 4 °C for 30 min). The measurement of LPS concentration was conducted by means of Pierce LAL chromogenic endotoxin quantification kit (Life Technologies, Carlsbad, CA) and according to the manufacturer instructions. *In vitro* release of the LPS from the particles was investigated in PBS (pH = 7.4) at 37 °C. Briefly, 1 mL of nanoparticle suspension was centrifuged at 15,000 *g* at 4 °C for 30 min, and the supernatant was removed. The nanoparticle pellet was then resuspended in 10 mL of release medium, kept in a shaking water bath (70 rpm) where samples were drawn at specific intervals. The samples were then centrifuged at 15,000* g* for 30 min (at 4 °C), and the concentration of the free LPS was determined in the supernatant as previously explained.

### Interaction with the immune cells

4 × 10^5^ RAW 264.7 macrophages or JAWS II DCs were separately seeded in 24-well plates and left to adhere. The cells were subsequently incubated overnight with different concentrations of either LPS solution or corresponding amounts of LPS-NP suspension. A control set of experiments with LPS-free PLGA nanoparticles was also analogously conducted. The supernatant was used to determine the induction of different cytokines (TNF-α, IL-12, IL-1β, and IL-6) using enzyme-linked immunosorbent assay (ELISA) (eBioscience, Thermo Fisher Scientific, ‎Waltham, MA and BD Bioscience, Heidelberg, Germany) according to the manufacturer instructions. Toxicity of the particles for the macrophages/DCs was assessed by means of the MTT assay.

CLSM was used to visualize the interplay of the LPS molecules and the nanoparticles following interaction with the immune cells. To this end, 2 × 10^5^ RAW264.7 cells were seeded in monolayer on coverslips and were incubated overnight for adherence. The cells were then treated overnight with Nile Red loaded FITC-conjugated LPS-NPs (final LPS concentration 10 µg/mL). The cells were then fixed with 4% paraformaldehyde, and the nuclei were stained with 300 nM DAPI (Sigma-Aldrich). The samples were mounted on slides and examined using Nikon Eclipse Ti CLSM (Nikon Cooperation Inc., Tokyo, Japan). Colocalization of FITC-conjugated LPS and Nile Red-loaded nanoparticles was determined in terms of Pearson correlation and Mandel’s overlap (for 50 cells) using Nikon NIS Elements Advanced Research software (Nikon Cooperation Inc., Tokyo, Japan).

To assess the efficiency of TLR4 activation, the cellular concentration of NF-*κ*Bp65 was determined in cell lysates 6 h after the incubation of RAW 264.7 macrophages with LPS or LPS-NP. Briefly, 10^7^ cells were seeded in 25 cm^2^ culture flasks and treated with two different concentrations of LPS solution or LPS-NP suspension (10 and 30 µg/mL). These concentrations were selected as they were associated with more than 80% of cell recovery after 6 h of incubation. Following the incubation, the supernatant was removed, the cells were washed twice with cold PBS, and 5 × 10^6^ cells were harvested for cellular extraction. To this end, cells were lysed in cell extraction buffer (Life Technologies) supplemented with 1 mM PMSF (Life Technologies), and protease inhibitor cocktail (Sigma-Aldrich) for 30 min on ice with vortexing at high speed at 10-min intervals. Concentration of NF-*κ*Bp65 was thereafter measured within the cellular extract using ELISA (Life Technologies) and according to the manufacturer instructions. To determine the impact of TLR4-independent NF-*κ*B activation, a control set of experiments was analogously conducted on the macrophages whose TLR4 signaling pathway had been blocked prior to the treatment with LPS/LPS-NP. The blockage of TLR4 signaling was achieved through 6 h pre-incubation with CLI-095 (InvivoGen, San Diego, CA). This enabled the investigation of the impact of impurities (e.g. nucleic acid impurities) as well as the potential TLR4-independent pro-inflammatory properties of the polymer matrix.

### Co-culture experiments

To compare the effect of the free and particle-bound LPS in a mixture of primary immune cells, 2 × 10^5^ adhered C26 cells were incubated together with 5 × 10^6^ freshly isolated splenocytes, followed by overnight treatment with two different concentrations of LPS/LPS-NP (30 and 10 µg/mL). The next day, the immunogenic cell death was evaluated through the quantification of caspase 3 levels within the cell debris using EnzChek^®^ Caspase-3 Assay Kit #2, Z-DEVD-R110 substrate (Life Technologies). Additionally, the induction of apoptosis in the tumor cells was further confirmed by flow cytometry. Briefly, the supernatant was removed, the cells were washed twice with PBS, and the tumor cells were isolated from splenocytes by Percoll (GE Healthcare, ‎Chicago, IL) gradient centrifugation according to a protocol described elsewhere (Liu et al., 2013). Tumor cells were subsequently resuspended in 1 mL of Annexin V binding buffer (BD Bioscience), labeled with FITC-conjugated Annexin V and PI (BD Bioscience) according to the manufacturer instructions, and examined by flow cytometry (FACSCalibur^TM^, BD Bioscience, Heidelberg, Germany). The results were analyzed using FlowJo version v10.1r7 (FlowJo LLC, Ashland, OR), and the quadrants were set based on untreated C26 cells cultured without the splenocytes. The induction of early apoptosis was ascertained based on the calculation of the number of Annexin V positive/PI negative cells in three independent experiments.

### In vivo therapeutic efficiency

*In vivo* therapeutic efficiency was assessed in tumor-bearing mice. Briefly, 3 × 10^5^ C26 cells were dispersed in 100 µL of PBS and subcutaneously injected into the lower right flank of 6-week-old male BALB/c mice. Treatment was initialized on the 10th post-injection day. LPS was injected biweekly in two different doses (100 and 1000 µg/mL) either as solution or as LPS-NP (freshly prepared) in three corners around the tumor (total injected volume of 100 µL per animal per dose). Tumor volume was measured as an indicator of the therapeutic response (volume = (width)^2^ × length/2). The animals were sacrificed once the tumor surpassed a volume of 1000 mm^3^. The weight of the animals was also biweekly controlled. Surviving mice were thrice (every 80 d) rechallenged with an injection of C26 cells in their left flank. Tumor growth was periodically monitored, and when necessary, tumor volume was calculated.

To check the possibility of cross-immunity, the experiments were repeated on a new set of animals, where a complete remission of the syngeneic colorectal cancer was observed under the effect of treatment with LPS-NP at both concentrations and LPS solution at 100 μg/mL. On the day 85 after the initial inoculation of the C26 cells, the animals were challenged with the injection of 5 × 10^5^ GL261 cells in their left flank. Tumor inoculation was analogously conducted in an untreated control group, which had not been involved within the original challenge. Tumor growth was periodically monitored and tumor volume was calculated. The animals were sacrificed once the tumor volume exceeded 1000 mm^3^.

In order to demonstrate that the higher tolerability of the LPS-NP is in fact due to the incorporation of the LPS molecules within the nanoparticle structure, tumor-bearing animals were injected with the LPS-NP suspension (1000 µg/mL) prepared 3 d prior to the treatment, where a significant amount of the LPS had been released from the particle surface (more than 90%). The animals were controlled in terms of the occurrence of internal or external necrosis at or around the site of injection. The results were compared to those obtained for the animals treated with LPS solution and fresh nanoparticle suspension (1000 µg/mL).

To compare the impact of the localized and none-localized induction of the immune response, the animals inoculated with C26 cells in their right flank were injected with two concentrations of LPS-NP (100 and 1000 µg/mL) in their left flank. The injections were carried out in a bi-weekly manner, and the animals were sacrificed once the tumor volume exceeded 1000 mm^3^.

### Investigation of the 24 h post-injection systemic TNF-α and LPS concentration

Serum concentrations of TNF-α and LPS were determined 24 h after the peritumoral injection of the first dose of PBS (control), LPS or LPS-NP at high concentrations (1000 µg/mL) using ELISA (eBioscience) and LAL chromogenic endotoxin quantification assay, respectively.

### Microscopic evaluation of the tumor cross-sections

For tracking the LPS penetration (both free and nanoparticle-bound) into the tumor after injection, FITC-labeled LPS was utilized in the preparation of both the solution and nanoparticles, which were injected in three corners around the tumor. Animals were sacrificed either 1 or 24 h after administration and the tumor was taken for microscopic examination directly using inverted Nikon Eclipse Ti CLSM (Nikon Cooperation Inc., Tokyo, Japan). The green fluorescence of FITC was detected following excitation with an argon laser (excitation wavelength at 488 nm), and by subsequent collection of the fluorescence signals using bandpass filters of 525 nm. The autofluorescence of the tissue was eliminated against an untreated control sample. The samples were examined either for 2D surface view or optically sectioned into the Z axis to get a 3D reconstruction of the tissue section. Laser power (5%), pinhole size (0.1), and scanning speed (one frame per second) were kept constant for all experiments.

To investigate the intratumoral infiltration of CD14^+^ cells, tumors were isolated from the control mice as well as those treated with multiple doses of 100 µg/mL LPS or LPS-NP. The tumor was fixed in 4% paraformaldehyde for 24 h, embedded in paraffin, and vertically cut in 4 µm cross-sections, which were subsequently stained with 10 µg/mL FITC-labeled anti-mouse CD14 (eBioscience) and 300 nM DAPI (Sigma-Aldrich) overnight, in a humidified chamber at 4 °C. The cross-sections were mounted on slides and examined using inverted Nikon Eclipse Ti CLSM as previously described. The extent of intratumoral infiltration of CD14 expressing cells was semi-quantitatively assessed by measuring the average percentage of the stained surface area in 10 different fields obtained from various areas of three tumor cross-sections using Image J^®^ software (NIH Image J system, Bethesda, MD).

### Statistical analysis

Statistical analysis of the *in vitro* experiments was performed with GraphPad InStat3 (GraphPad Software, La Jolla, CA). The comparison of data points with the control was conducted using One-way Analysis of Variance (ANOVA) followed by Dunnett Multiple Comparison test. Unpaired t-test with Welch correction was used to compare the cytokine induction profiles. Significance levels included *p* < .05 (*), *p* < .01 (**), and *p* < .001 (***). *In vitro* dose-response fitting and LC50 calculation was performed using Origin Lab^®^ version 8 (OriginLab Corporation, Northampton, MA).

## Results

### Nanoparticle characterization

**Figure 1.**
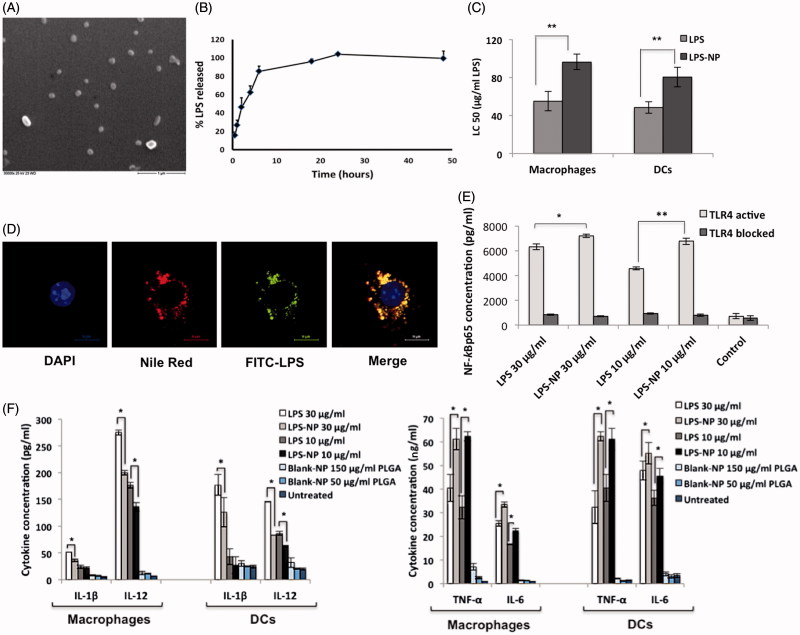
*In vitro* characterization of LPS-NP and the associated pro-inflammatory properties. A) Scanning electron microscopic examination of the LPS-NP morphology (scale bar represents 1 µm). B) LPS release from the nanoparticles under sink conditions. C) LC50 values calculated based on the dose-response fitting of cell survival values following the overnight incubation of 4 × 10^5^ RAW 264.7 macrophages or JAWS II DCs with different concentrations of LPS and LPS-NP. D) Co-localization of LPS and nanoparticles following overnight incubation of macrophages with LPS-NP (Pearson’s correlation for 50 cells: 0.8666 ± 0.03964, Mander’s overlap for 50 cells: 0.8693 ± 0.0396). Blue, red, green, and yellow colors represent the cell nucleus, nanoparticles, LPS, and the co-localization of the LPS and nanoparticles, respectively. Scale bars represent 10 µm. E) Induction of NF-κBp65 in 5 × 10^6^ RAW 264.7 macrophages following 6 h incubation with LPS/LPS-NP. RAW 264.7 cells with TLR4 blocked signaling have been used as control to enable the determination of the TLR4-independent induction of NF-κBp65, potentially related to the PLGA matrix or impurities. F) Induction of pro-inflammatory cytokines following the overnight incubation of 4 × 10^5^ RAW 264.7 macrophages or JAWS II DCs with LPS, LPS-NP, and blank PLGA nanoparticles with polymer concentrations corresponding to those of LPS-NP.

The prepared nanoparticles had an effective diameter of 155 ± 20 nm, with an acceptable polydispersity index (PDI) of 0.123 ± 0.007. Given the negative charge of both the matrix and the LPS molecules (Schromm et al., 1998; Mura et al., 2011), the overall zeta potential of the particles was predictably negative (–15 ± 0.2). The percentage of the decorated LPS was equal to 69 ± 4%. The particles had a spherical morphology with a smooth surface (Figure 1(A)). The observed particle size in scanning electron microscopic (SEM) pictures was in concurrence with the results obtained through photon correlation spectroscopy. LPS-free PLGA nanoparticles had a size 135 ± 10, an acceptable PDI of 0.125 ± 0.035, and zeta potential of −7 ± 0.2.

Figure 1(B) shows the time-dependent *in vitro* release of LPS from the nanoparticles. Due to their superficial localization on the particle matrix, LPS molecules were released in a burst manner mainly within the first 8 h of the experiments when exposed to sink aqueous conditions. It should be noted, however, that the *in vivo* release profile of the LPS would be significantly retarded given the subcutaneous injection, which hinders the particles from direct exposure to sink conditions.

### Interaction with macrophages and DCs

The toxicity of the LPS and LPS-NP was compared through the calculation of LC50 values for RAW 264.7 macrophages and JAWS II dendritic cells (DCs) (Figure 1(C)). As observed, compared to LPS solution, LPS-NP exhibits significantly lower toxicity for both cell lines. Blank LPS-free PLGA nanoparticles showed no significant toxicity for the cell lines within the used concentration range.

The interaction of LPS, nanoparticles, and macrophages was visualized through confocal laser scanning microscopy (CLSM) following overnight incubation (Figure 1(D)). The results demonstrated a high level of nanoparticle internalization by the macrophages, as well as a high colocalization of the LPS and the nanoparticles following uptake (Pearson’s correlation for 50 cells: 0.8666 ± 0.0396, Mander’s overlap for 50 cells: 0.8693 ± 0.0396). This indicated that due to the fast uptake of the particles by macrophages, a significant part of the decorated LPS remained incorporated within the nanoparticle structure despite the burst release of the LPS molecules with the first 8 h.

Compared to free LPS solution, LPS-NP was shown to induce higher levels of NF-*κ*Bp65 in RAW 264.7 macrophages (Figure 1(E)). Incubation with TLR4-blocked macrophages demonstrated the pro-inflammatory properties of the particles to be TLR4-mediated, while the TLR4-independent NF-*κ*B induction related to the nucleic acid impurities or the particle matrix was shown to be relatively negligible.

Figure 1(F) depicts the induction of pro-inflammatory cytokines in macrophages and DCs following stimulation with LPS/LPS-NP. As observed, in both cell lines, LPS-NP are stronger inducers of TNF-α and IL-6, whereas pure LPS is a more potent stimulator of IL-12 and IL-1β (at higher concentration). The PLGA blank particles accounted for a slight induction of pro-inflammatory cytokines, TNF-α and IL-6 paramount, though the induction was far less significant relative to the LPS and LPS-NP.

### Induction of immunogenic cell death in tumor-splenocyte co-cultures

**Figure 2.**
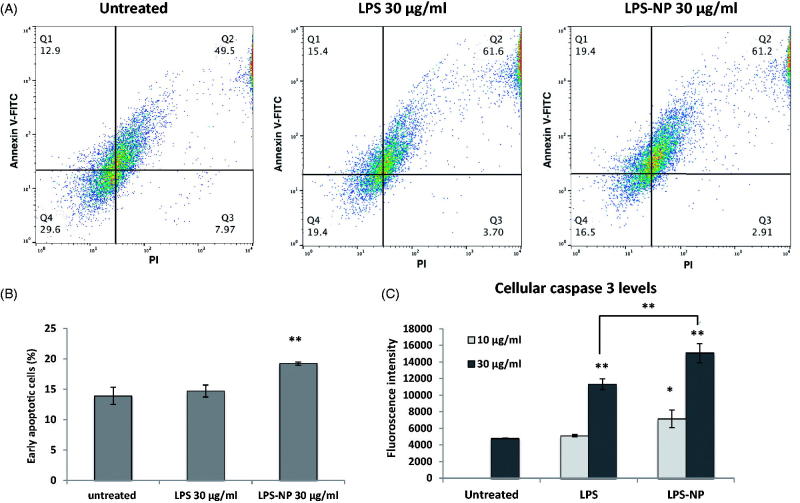
Comparison of the immunotherapeutic potentials of LPS and LPS-NP in tumor-splenocyte co-cultures. Apoptosis induction in C26-splenocyte co-culture treated overnight with LPS or LPS-NP investigated through A) flow cytometric analysis of Annexin V and PI-stained C26 cells (quadrants were set based on untreated C26 cells cultured without the splenocytes), B) the resulted percentage of early apoptosis cells, and C) quantification of caspase 3 levels within the cellular extracts.

A co-culture of tumor cells and splenocytes was used to compare the antitumor effect of free and particle-bound LPS on a mixture of primary immune cells. Though a rich source of lymphocytes, splenocytes also contain a significant number of monocytes such as macrophages and DCs (Bronte & Pittet, 2013). The induction of apoptosis was determined both through the direct measurement of the caspase 3 levels, and through the flow cytometric evaluation of the tumor cells following Annexin V and PI staining. Overnight treatment of the co-cultures with both LPS and LPS-NP resulted in a significant increase of the cellular caspase 3 levels compared to the untreated control, though the increment was significantly higher in case of LPS-NP (Figure 2(C)). For LPS solution, caspase 3 induction was only pronounced at higher concentration (30 µg/mL), whereas LPS-NP resulted in a significant increase of caspase 3 at both tested concentrations (10 and 30 µg/mL). Similarly, a significant increase of the early apoptotic cells was observed following the flow cytometric analysis of the tumor cell populations, which was more pronounced in case of the cells treated with 30 µg/mL of LPS-NP (Figure 2(A,B)).

### In vivo therapeutic efficiency

#### Lack of localized necrosis in case of highly concentrated LPS-NP (1000 µg/mL)

**Figure 3.**
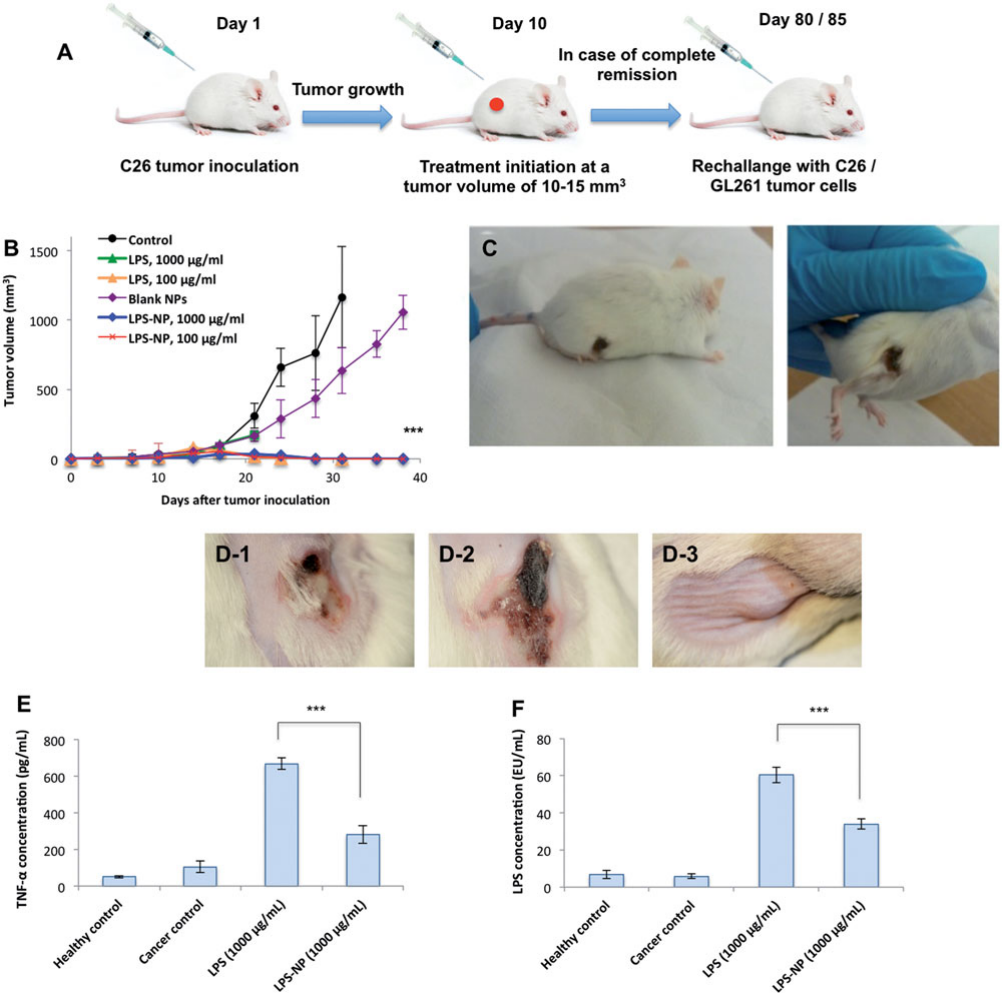
Comparison of the immunotherapeutic efficiency and tolerability of LPS and LPS-NP *in vivo*. A) Schematic of the animal trials, B) complete tumor regression was observed after the administration of 4–5 doses of LPS (100 µg/mL) and LPS-NP (both 1000 and 100 µg/mL), while C) severe necrosis occurred in the animals treated with 1000 µg/mL LPS solution. D) Localized necrosis was observed following the peritumoral injection of 1000 µg/mL LPS solution (D-1) and LPS-NP prepared three days prior to the treatment with more than 90% released LPS (D-2). No necrosis was observed in the group treated with freshly prepared LPS-NP (D-3), which signifies the LPS decoration to be the main reason for lack of post-injection necrosis. Serum TNF-α (E) and LPS (F) levels were measured 24 h following the peritumoral injection of the first dose of LPS/LPS-NP at high concentrations (1000 µg/mL). Data are also presented for healthy and cancerous (PBS-treated) control animals. Significantly higher concentration of both TNF-α and LPS was observed in the animals treated with LPS solution, as evidence of the necrosis being localized Shwartzman phenomenon. The results are presented as the mean ± SD of the experiments on five different animals per each group.

The general schematic of the animal trials is depicted in Figure 3(A). Biweekly treatment of the tumor-bearing mice with peritumoral injections of 100 µg/mL LPS, 100 µg/mL LPS-NP, and 1000 µg/mL LPS-NP resulted in complete tumor regression after the administration of 4–5 doses (Figure 3(B)). Treatment with 1000 µg/mL LPS solution, however, was intolerable for the mice resulting in severely localized necrosis and hampering the continuation of the experiments on the animals in this group (Figure 3(C)).

In order to assure that the lack of localized necrosis in case of high concentration of LPS-NP (1000 µg/mL) indeed pertains to the incorporation of the LPS molecules within the nanoparticle structure, we explored the impact of nanoparticle age upon the *in vivo* therapeutic side effects. Given the superficial localization of the LPS, as indicated by the release studies, LPS molecules tend to release from the nanoparticle surface overtime. Consequently, a considerable amount of LPS would be available as free molecules several days after nanoparticle preparation. We, therefore, injected the animal’s peritumorally with 1000 µg/mL LPS solution, freshly prepared LPS-NP, and the LPS-NP prepared 3 d prior to the experiments (with more than 90% release of the incorporated LPS). Interestingly, unlike freshly prepared nanoparticle suspension, both the LPS solution and old nanoparticle suspension resulted in localized necrosis (Figure 3(D-1,D-2)). Treatment with freshly prepared nanoparticle suspension, however, was very well tolerated and no case of localized necrosis was observed (Figure 3(D-3)).

To verify the possibility of the localized necrosis being a byproduct of Shwartzman phenomenon, serum levels of TNF-α and LPS were determined 24 h after the first peritumoral injection of LPS and LPS-NP (1000 µg/mL) (Figure 3(E,F)). As observed, treatment with LPS solution accounted for significantly higher systemic concentrations of both TNF-α and LPS.

#### Higher intratumoral deposition of the LPS-NP at lower concentration (100 µg/mL)

**Figure 4.**
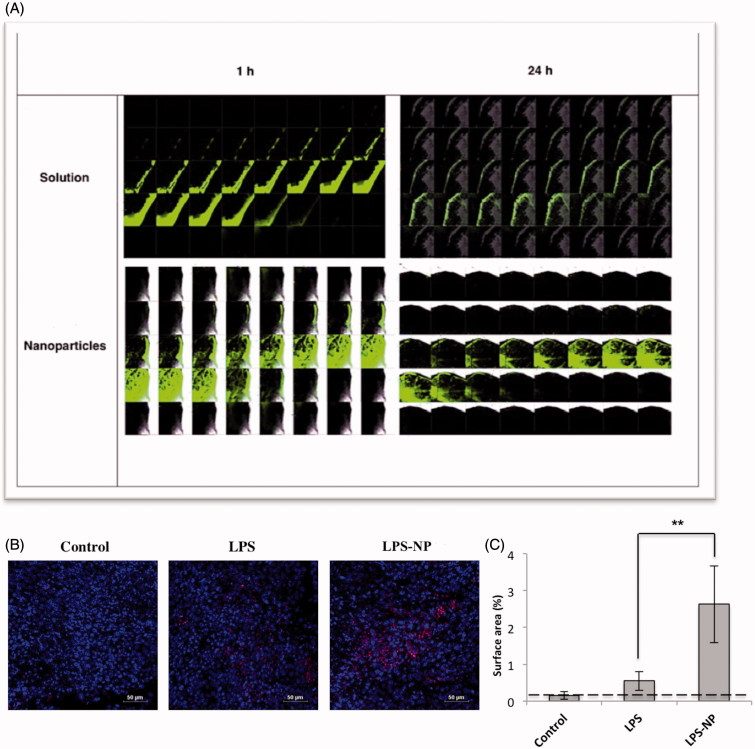
Microscopic evaluation of the tumor cross-sections. A) Intratumoral LPS penetration and deposition 1 and 24 h after the peritumoral injection of LPS/LPS-NP (100 µg/mL). The width of each individual field and the distance between two consecutive cross sections are 258 and 5 μm, respectively. B) Intratumoral infiltration of macrophages (CD14 + cells) in the animals treated with PBS (control), and 100 µg/mL of LPS or LPS-NP. CD14 + cells are shown in pink while the nuclei are dyed blue. Scale bars represent 10 µm. C) Analysis of the percentage of the CD14 + stained surface area from 10 independent fields captured from various areas of three tumor cross-sections indicating significantly higher intratumoral infiltration of the CD14 + cells in the animals treated with LPS-NP compared to the other groups.

Although both LPS solution and LPS-NP resulted in complete remission at the lower concentration (100 µg/mL), a significantly higher intratumoral penetration and deposition of the FITC-conjugated LPS was observed in the animals treated with LPS-NP. As observed in Figure 4(A), a stronger fluorescence was detected both 1 and 24 h after the injection. Additionally, higher intratumoral infiltration of CD14^+^ cells, as potential carriers of the LPS-NP, was observed in the tumor cross-sections obtained from the animals treated with LPS-NP compared to the LPS-treated and control groups (Figure 4(B,C)).

#### Rechallenge and cross-immunity studies

**Figure 5.**
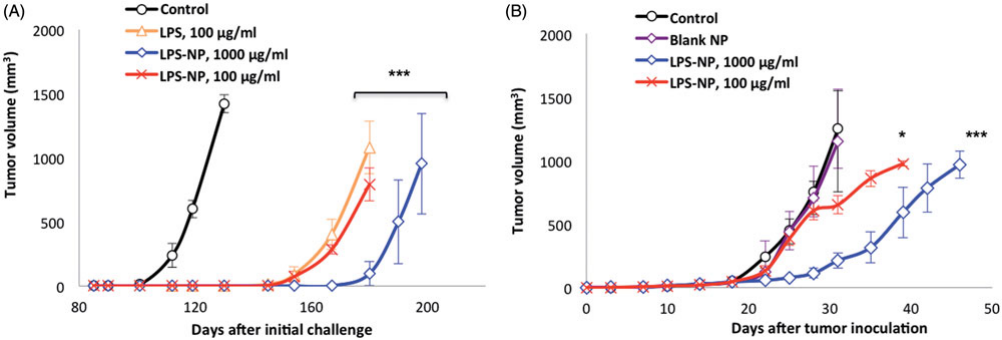
Cross-immunity and the importance of the localized injection of the nanoparticles close to the tumor site. A) Investigation of cross-immunity through the injection of GL261 cells in the left flank of the mice previously recovered from syngeneic colorectal cancer and B) tumor growth following the inoculation of C26 cells in the right flank and biweekly injection of LPS-NP in the left flank of the mice.

The follow-up monitoring of the surviving animals within the initial set of experiments revealed that in the three groups with full recovery (LPS 100, LPS-NP 100, and 1000 µg/mL), only one animal per group had recurring tumor. Following remission, surviving animals were rechallenged three times with subcutaneous injections of C26 cells in their left flank (every 80 d). Merely one case of tumor growth was observed within the group having been treated with 100 µg/mL LPS solution, while no recurrence occurred in the nanoparticle-treated groups. Additionally, some degrees of cross-immunity were observed, for the growth of GL261 xenograft tumor was significantly delayed in the animals with regressed colorectal tumor compared to the unchallenged control group. The longest delay was observed in case of the animals having been treated with high concentration (1000 µg/mL) of LPS-NP (Figure 5(A)). The observed cross-immunity might pertain to the similarity of some tumor antigens presented by both cell lines. For instance, serum levels of carcinoembryonic antigen (CEA) have been reported to significantly increase in patients suffering from both colorectal cancer (Su et al., 2012) and glioma (Suzuki & Tanaka, 1980).

#### Importance of the localized activation of the immune response

When injected in the opposite flank, biweekly treatment with LPS-NP (in particular at 1000 µg/mL) could significantly retard the tumor growth, though unlike the peritumoral injection, no case of complete remission was observed. This signified the essentiality of the localized administration of the immunotherapeutic system for maximized tumor antigen identification, processing, and presentation (Figure 5(B)).

## Discussion

The success of LPS-based immunotherapy is hampered by the associated side effects, which restrict both the dosage escalation and chronic dosing possibilities. Notwithstanding the initial promise of tumor regression, systemic administration of LPS, and similar immunomodulators is fraught with immunotoxicological concerns limiting the administrable dosage to a few ng per kg body weight (Garay et al., 2007). Although various approaches, detailed in the introduction, have been developed to address such issues, maintaining the compound’s immunostimulatory properties while reducing the associated side effects still poses a challenge.

Herein, we sought to develop a simple nanoparticle formulation to address this old problem, and to improve the efficiency and alleviate the adverse effects of LPS-based immunotherapy in cancer. Rather than regarding the LPS molecules as a cargo to be loaded within the nanoparticle structure (which has been reported in a few studies for vaccination purposes (Gómez et al., 2008; Demento et al., 2009)), we decided to exploit the surface-active properties of the LPS molecules (Aurell & Wistrom, 1998) for their incorporation as a building unit of the nanoparticle structure. This would eliminate the need for the use of further (potentially efficiency compromising) surface-active stabilizers in the system, while ensuring the localization of the amphiphilic LPS molecules upon the interface of the nanoparticles and the surrounding aqueous medium (Heinz et al., 2017). The latter was believed to influence the conduct of the LPS molecules at the cellular and molecular levels. On one hand, the superficial decoration of the LPS molecules can endow the particles pathogen mimicking properties, while the size of the system can enhance their ‘visibility’ to the cells of the innate immunity (Petersen et al., 2011; Beletskii et al., 2014). On the other, when injected under non-sink conditions (e.g. subcutaneously or intramuscularly), the non-covalent binding of the LPS molecules would allow for a significant formulation-mediated control over their systemic release. This can in turn modulate the side effect of the treatment by balancing the rates of the systemic exposure of the monocytes to the circulating LPS and the LPS clearance from the body (Satoh et al., 2008). In fact, both the immunostimulatory properties of the nanoparticles and the release of the LPS therefrom are optimizable through the manipulation of the particles’ physicochemical characteristics (Dobrovolskaia & McNeil, 2007; Mottram et al., 2007), and a wise selection of the nanoparticle composition, both in terms of the matrix and the hydrophilic-lipophilic balance (HLB) of the decorating LPS molecules (Aurell & Wistrom, 1998; Maggio, 2012; Shakya & Nandakumar, 2013; Jiao et al., 2014). With such possibilities in mind, PLGA-based LPS-NP were formulated and investigated from a new perspective, wherein the (immunotherapeutic) cargos can actively participate within the construction of their nanoparticulate carriers, and the resultant interactions there between can determine the overall efficiency and tolerability of the structurally incorporated payload. PLGA was selected as the polymeric matrix for the preparation of these primary set of particles due to its favorable characteristics including biodegradability, biocompatibility, and safety, which has accounted for its approval for clinical administration (Mundargi et al., 2008). Nonetheless, further polymeric/lipid-based matrices of higher immunogenicity are also exploitable for the purpose of particle design.

According to the results of the cell culture experiments, decoration of the LPS molecules on the nanoparticle surface accounted for a reduced toxicity and an altered cytokine induction profile. Within the context of the latter, induction of TNF-α and IL-6 was higher under the effect of LPS-NP, while LPS was a more potent stimulator of IL-12 and IL-1β. Since the overall pro-inflammatory properties of both LPS and LPS-NP were to a similar extent TLR4-mediated, the increased size of the system in case of the particle-bound LPS seems to have compromised the intracellular immune response induced under the effect of TLR4 activation. The altered cytokine induction pattern is not much related to the net pro-inflammatory properties of the LPS-free PLGA matrix, which merely resulted in relatively low levels of TNF-α induction. Despite the difference in cytokine induction patterns, LPS-NP were associated with stronger overall immunostimulatory potentials, since they accounted for a higher induction of NF-*κ*B compared to the LPS solution. In line with these observations, the particles were also revealed to be superior inducers of apoptosis in C26-splenocyte co-cultures, as evident from the higher post-incubation cellular caspase 3 levels and the significant increase in the number of early apoptotic tumor cells. It should be noted that due to the short duration of the co-culture experiments, the observed antitumor impact is mainly related to the activation of the innate immunity, in particular the induction of TNF-α, which is more pronounced in case of LPS-NP.

*In vivo*, peritumoral injection of 1000 µg/mL of LPS-NP accounted for tumor regression in all animals, while the same LPS dosage as solution led to severe localized necrosis. The necrosis did not manifest on the day of treatment, but commenced to appear 24–48 h following the first LPS injection. To ensure that the lack of necrosis in animals treated with LPS-NP is in fact due to the incorporation of the LPS within the nanoparticle structure, a group of animals were injected with the nanoparticle formulation prepared 3 d prior to the injection, in which a significant part of the LPS (more than 90%) had been already released from the nanoparticle surface. Interestingly, unlike the freshly prepared nanoparticle formulation, treatment with old LPS-NP suspension led to necrosis similar to that caused by pure LPS solution. We believe that the observed necrosis in case of high-concentration LPS solution might pertain to Shwartzman reactions at the site of injection. An excellent *in vivo* correlate of the septic shock, Shwartzman phenomenon can manifest as a local or generalized reaction (Aguillon et al., 1996). The former is induced through the localized injection of a preparing agent (often LPS) and the intravenous injection of a provoking agent (an agent which can initiate intravascular coagulation) (Hjort & Rapaport, 1965) with 18–24 h interval, while injection at other time points will not trigger the reaction (Aguillon et al., 1996). Although LPS is the most prevalent provoking agent for this purpose, TNF-α has been introduced as an appropriate candidate due to its ability to impair anticoagulant processes (Aderka, 1991). Both localized and systemic increase of TNF-α concentration 24 h after the LPS sensitization has been shown to cause hemorrhagic necrosis in mice, and the reaction depends on the synergistic effect between TNF-α (also endogenous TNF-α) and the sensitizing endotoxin (Rothstein & Schreiber, 1988). By the same virtue, high systemic concentration(s) of TNF-α and/or LPS 18–24 h after the localized injection of the LPS might act (perhaps synergistically) as a provoking agent and trigger the reaction. This is further confirmed by the fact that the occurrence of localized hemorrhagic necrosis in mice has been also reported following a single intradermal injection of 30–80 µg of LPS (Ishikawa et al., 1991). Herein, we found significantly higher serum TNF-α and LPS concentrations 24 h after the injection of the LPS solution at high concentration (1000 µg/mL) as evidence of such a possibility. The delayed onset of the necrosis further supports the abovementioned hypothesis. Therefore, the ability of the nanoparticles to inhibit the occurrence of local necrosis might pertain to the modulation of the TNF-α induction and the LPS systemic release patterns, altering their systemic concentrations to lower levels than the threshold of Shwartzman reactions at the critical time span of 18–24 h post-injection. Further investigation of the pathological basis of the reaction is required to better understand the underlying biological phenomena. Regardless, as highlighted by the findings of the *in vivo* experiments, LPS incorporation within the nanoparticle structure significantly enhances the overall tolerability of the system, thus offering the possibility of increasing the maximum administrable dosage. Since the efficiency of LPS-based immunotherapy is concentration dependent, the dose escalation possibility can highly improve the chances of positive therapeutic outcomes.

When injected at lower concentration (100 µg/mL), both LPS solution and LPS-NP resulted in complete tumor regression. Nevertheless, particle-bound LPS was shown to have higher intratumoral penetration and deposition compared to its free counterpart. These findings were initially quite surprising, since in theory, nanoparticles are supposed to have a lower intratumoral penetrability compared to pure molecules, particularly considering the dense package of the tumor cells, and the presence of the collagenated extracellular matrix (MacEwan et al., 2010; Nichols & Bae, 2012). However, a possible explanation for this controversy can be the higher uptake of the nanoparticle-bound LPS by the tumor-infiltrating immune cells (in particular APCs). As previously debated, by virtue of the larger size of the system, particle-bound LPS molecules have higher ‘visibility’ to the cells of innate immunity, and consequently a higher uptake thereby. These potential carriers of the particle-bound LPS are expected to have a high expression of CD14, a monocyte marker associated with the LPS cellular uptake and TLR4 activation (Zanoni et al., 2011). Therefore, the greater intratumoral infiltration of CD14^+^ cells within the nanoparticle-treated group is in concurrence with the abovementioned hypothesis and further confirms the pathogen-mimicking properties of the particles.

As reported for several other TLR ligands (Heckelsmiller et al., 2002; Nierkens et al., 2009), localized injection close to the tumor site seems crucial to obtain maximum therapeutic efficiency. This study is further confirmatory; as the subcutaneous injection of the nanoparticles in the opposite flank could merely, though significantly, retard the tumor growth. In case of the LPS immunotherapy, the majority of the previous studies have focused on the intravenous or intratumoral administration (Engelhardt et al., 1991; Otto et al., 1996; Chicoine et al., 2001). As debated, systemic LPS injection is associated with strong inflammatory reactions limiting the injectable dosage to several ng per kg body weight (Garay et al., 2007). Intratumoral injection, on the other hand, is inappropriate for particulate systems due to their limited intratumoral penetrability (MacEwan et al., 2010; Allhenn et al., 2013). The peritumoral injection redirects the immune response toward the tumor site, where a large reservoir of tumor antigens is in hand, while alleviating the undesirable systemic side effects. While peritumoral injection is of particular interest for superficial cancers, such as melanoma, injection of the particles in the vicinity of other solid tumors is often clinically plausible. Otherwise, and as a further advantage, the carrier properties of the developed LPS-NP would allow for the incorporation of tumor antigens that can properly orient the activated immune response. Within the context of this study and using the peritumoral injection paradigm, complete remission could be achieved with lower amounts of LPS compared to those reported in previous studies having used systemic or intratumoral injections (Goto et al., 1996; Chicoine et al., 2001). It should be noted that factors, such as the onset of treatment as well as the type and batch-to-batch variation of the LPS (which impact its surface-active properties) are important determinants of the therapeutic outcome.

In all, the findings confirmed our initial hypothesis postulating that the use of LPS molecules as building units of the nanoparticle structure can improve the outcome of LPS-based immunotherapy in cancer. While the resultant alleviation of the side effects at high concentrations allows for a significant escalation of the administrable dosage, the improved intratumoral deposition of the particle-bound LPS molecules signifies their more effective interaction with the cells of the innate immunity by virtue of their pathogen-mimicking properties. Of course, the results of this study can be extrapolated to further therapeutic payloads with structural properties that render them appropriate for serving as building units of their nanoparticulate carriers.

## Conclusions

Incorporation of LPS in the nanoparticle structure was shown to improve the therapeutic outcome of LPS-based active immunotherapy in cancer. The particles modulated the cytokine induction pattern, possessed an enhanced intratumoral deposition, resulted in the elimination of the localized side effects at high concentrations, and offered the possibility of dose escalation. These findings open door to new possibilities for the wise and purposeful design of further LPS-based immunotherapeutic nanoparticulate systems, benefiting from various advantages of particle engineering and formulation, including the use of different immunostimulatory matrices, selection of LPS molecules with different HLB values, and manipulation of the particles’ physicochemical characteristics. Furthermore, based on their physicochemical properties and molecular structures, other therapeutic cargos can be exploited as the building units of their nanoparticulate carriers, shifting thereby the role of nanoparticles from mere drug carriers to systems with potent therapeutic properties.
